# Impact of a Food Skills Course with a Teaching Kitchen on Dietary and Cooking Self-Efficacy and Behaviors among College Students

**DOI:** 10.3390/nu16050585

**Published:** 2024-02-21

**Authors:** Caitlin D. French, Alexander Gomez-Lara, Arianna Hee, Akshara Shankar, Nayoung Song, Monserrath Campos, Mikelle McCoin, Susana L. Matias

**Affiliations:** Department of Nutritional Sciences & Toxicology, University of California Berkeley, Berkeley, CA 94720, USA; cdfrench@berkeley.edu (C.D.F.); argomez@berkeley.edu (A.G.-L.); ariannah@berkeley.edu (A.H.); akshara.shankar12@berkeley.edu (A.S.); nayoung.song@berkeley.edu (N.S.); monsec@berkeley.edu (M.C.); mikellem@berkeley.edu (M.M.)

**Keywords:** teaching kitchen, nutrition curriculum, college students, young adults, cooking skills, self-efficacy, dietary intake, fruits and vegetables

## Abstract

College students may face barriers to eating healthy foods. Educational interventions providing practical knowledge and skills may help students to overcome financial barriers or other barriers to acquiring, preparing, and consuming healthy foods. We evaluated the association between participation in a semester-long food skills course with an interactive teaching kitchen and dietary and cooking self-efficacy and behaviors. Participants were recruited from course enrollees (intervention) and the general student population (comparison). We assessed differences in pre–post changes in the outcomes between groups using the propensity score weighting and mixed effects linear or Poisson regression. Course participation was associated with improved self-efficacy around cooking (group × time β-coefficient [SE]: 3.25 [0.57], *p* < 0.0001) and fruit (6.33 [1.19], *p* < 0.0001), vegetable (5.43 [1.42], *p* = 0.0002), and whole grain (5.83 [1.40], *p* < 0.0001) consumption. Course participants reported smaller pre–post decreases in vegetable consumption compared to non-participants (0.35 [0.16], *p* = 0.03), increased cooking frequency (0.22 [0.10], *p* = 0.03) and a decreased frequency of skipping meals (−0.47 [0.16], *p* = 0.003). There were no changes associated with the intervention in the consumption of fruit or whole grains, or in eating out frequency. Participation in a semester-long, personal food skills course with a teaching kitchen may improve self-efficacy, cooking, and vegetable consumption among college students.

## 1. Introduction

College students may face a number of challenges to healthy eating. For many who attend college during early adulthood, this stage of life may be characterized by moving away from home for the first time and gaining increased independence and control over their day-to-day activities, including meal acquisition or preparation. At the same time, the academic and monetary demands of attending college may place time and financial constraints on students’ ability to access nutritious food and make decisions around healthy eating behaviors [1,2]. For example, a study of diverse Midwestern community college and university students found that over a third to almost half of students reported time constraints affecting diet-related behaviors [3]. In addition, high rates of food insecurity have been reported among college students [4,5]; food insecurity is defined as having limited access to adequate foods due to a lack of money and other resources [6]. In 2015, the University of California Student Food Access and Security Study found that 42% of student respondents reported experiencing food insecurity, including 19% reporting very low food security [4]. Apart from time and resource constraints, other influential factors affecting cooking/eating behaviors among young adults and college students may include the physical food environment (e.g., campus dining halls, accessibility of grocery stores, etc.) [1,2], living on campus [7], and inadequate skills and knowledge around healthy food acquisition and preparation [1,7]. Students belonging to marginalized or underserved groups may be particularly affected by the above challenges. For example, students from racial/ethnic minority groups have reported unique challenges to developing and applying food literacy, such as a lack of access to culturally relevant food markets [2], and women and students of lower socioeconomic status reported more general time constraints [3]. Accordingly, a nationwide survey found that only 25.9% of undergraduate college students reported consuming at least three to four servings of fruits and vegetables per day, with 3.6% reporting consumption of five or more servings per day [8]. For reference, the 2020–2025 Dietary Guidelines for Americans recommends a combined four and a half servings of fruits and vegetables per day as part of a healthy dietary pattern (based on an energy intake of 2000 kilocalories per day) [9].

Observational research suggests that home food preparation behaviors, such as meal planning and cooking, and related skills are associated with healthier eating behaviors among young adults and college students [7,10]. For instance, young adults reporting more frequent food preparation behaviors (e.g., buying fresh vegetables, making a grocery list, preparing a meal for two or more people, etc.) consumed fast food less often and were more likely to consume five or more daily servings of fruits or vegetables [7]. Further, evidence from a U.S.-based cohort study tracking participants from adolescence to adulthood indicated that engagement in home food preparation in emerging adulthood, but not in adolescence, is predictive of a better diet quality in the mid-to-late twenties [11], pointing to early adulthood as a potentially optimal time to target interventions aimed at establishing long-term healthy dietary habits. Educational curricula focused on cooking and food acquisition skills may improve diet quality among college students, including by bolstering strategies to cope with food insecurity and increasing confidence around healthy eating [12]. In particular, teaching kitchens provide hands-on experiences for students to learn and practice new skills with their peers. Among college sophomores, they have been shown to increase cooking confidence more effectively than noninteractive cooking demonstrations [13]. Further, receipt of nutrition curricula with a hands-on cooking class component has been associated with increased reported fruit and vegetable consumption among healthcare professional trainees [14] and, paired with provision of vouchers for fresh produce, among SNAP participants [15].

While the evidence to date is promising and suggests overall positive changes in dietary behavior and cooking self-efficacy in response to cooking and home food preparation interventions, methodological limitations of previous research, including the lack of control or comparison groups in many studies, limit the strength of conclusions on the effectiveness of such interventions [16,17]. In addition, only a few studies have evaluated the effectiveness of educational interventions that include hands-on cooking classes among young adults or college students [12,13,18,19,20]. All of these studies, with the exception of the study by Matias et al. (2021) [12], included interventions that lasted 6 weeks or less. The current study therefore aimed to assess the impact of a semester-long food skills course with a teaching kitchen component on self-efficacy and behaviors related to healthy eating and cooking among undergraduate college students. This work extends our previous study, in which it was observed that cooking frequency, consumption of fruits and vegetables, and self-efficacy around these behaviors increased after participation in the course [12]. Here, we include a comparison group to isolate differences in pre–post changes among students enrolled in the course from other time-related changes.

## 2. Materials and Methods

### 2.1. Study Design and Participants

A quasi-experimental comparison study was carried out among undergraduate college students attending a large urban public university in northern California to assess the impact of a personal food skills (PFS) elective course, which was developed as part of the university’s efforts to address food insecurity among the student population. The intervention group included students enrolled in the PFS course during 1 of the 4 academic semesters occurring during the study period. Enrollment in the PFS course requires instructor approval and is based on prioritizing students at risk of food insecurity (see Table S1 in the Supplementary Materials for course screening questions). Each semester, a convenience sample of students not enrolled in the course were recruited from the general undergraduate student population to serve as the comparison group. Knowledge, skills, and behavior related to nutrition, food security, and food preparation were assessed at the beginning (first few weeks) of the semester, and again at the end (last few weeks) of the semester to compare changes in these outcomes in relation to course participation.

### 2.2. Intervention

The 14-week (semester-long) PFS course applies principles of Social Cognitive Theory [21] and aims to improve students’ knowledge, attitudes, self-efficacy, skills, and behaviors around food procurement and preparation. The details of the course content and structure have been previously described [12]. Briefly, the 2-unit elective course included weekly 50 min lectures and 2 h interactive cooking labs. The lectures covered topics related to basic nutrition (e.g., nutrients, their food sources, and calculation of personal requirements; reading food labels; mindful eating; etc.), food insecurity (including local food assistance resources), cooking (e.g., food storage and safety, cooking methods), and cost-saving techniques such as meal planning and food budgeting. The cooking labs included working in pairs to foster collaborative problem solving and reduce the perceived barriers related to cooking as students prepared easy-to-follow, quick, and affordable recipes, which emphasized plant-based meals but also included preparation of animal-based proteins. The students completed several projects to reinforce the course topics, including a 7 day, budgeted meal plan meeting specific meal and food-group criteria.

### 2.3. Study Procedures

The study was carried out over 4 semesters between August 2021 and May 2023. For recruitment into the intervention group, each student who enrolled in the PFS course was sent an initial email (plus up to 3 reminder emails) explaining the purpose of the study and inviting them to participate by following a link to an online, self-administered Qualtrics form containing an informed consent page followed by the baseline survey. In addition, at the beginning of each semester, a member of the research team visited the first lecture session of the PFS course and carried out recruitment via a brief announcement and paper handouts containing a QR code linking to the Qualtrics form. Fliers were also posted on the door of the teaching kitchen where cooking labs were held to recruit students enrolled in the course.

Recruitment of the comparison group included the same mechanisms that were used to advertise the PFS course to potentially interested students. These mechanisms consisted of distributing a flier and email invitation to various campus partners, including the undergraduate advisor of the department hosting the PFS course (the investigators’ home department) and representatives of other programs/centers serving students on campus who then distributed the invitation to students in their networks. Interested students followed the QR code or link to the consent form and baseline survey, where they indicated whether or not they were enrolled in the PFS course.

The intervention group determination was based on the final enrollment lists, which were provided to the investigators by the course instructor of record. Informed consent was obtained via the online baseline survey form, the first page of which consisted of the consent form and the corresponding survey item where participants indicated their consent. Only study participants who provided consent could proceed to the survey. After consenting, study eligibility was confirmed via two survey questions. Participants were eligible to participate in the study if they were (1) 18 years of age or older, and (2) an undergraduate student enrolled at the university. Survey respondents indicating ineligibility were redirected to the end of the survey. Those who were eligible, provided informed consent, and completed the survey were enrolled in the study.

The enrolled survey respondents in the intervention and comparison groups were invited by email to schedule an in-person baseline study visit to take 2 biometric measurements (i.e., skin carotenoid status and blood pressure), the results of which will be reported in a subsequent publication. During the last few weeks of the semester, study participants were sent emails (and up to 3 reminders) containing links to complete an endline survey, which measured the same outcomes as at baseline, and to schedule a second in-person study visit for biometric measurements. Comparison group participants were not contacted between the baseline and endline data collection points. For each online survey (baseline and endline) completed, respondents could choose to be entered into a random drawing to win 1 of 10 gift cards valued at 40 USD or 20 USD for intervention or comparison group participants, respectively. All study procedures were approved by the corresponding Institutional Review Board before implementation.

### 2.4. Measurement of Outcomes and Covariates

To assess the primary study outcomes of self-efficacy and behaviors related to healthy eating and cooking, we used a personal factors survey that was previously validated for use among college students [22]. The usual consumption of fruit, vegetables, and whole grains was assessed using 3 survey questions, which were adapted from 2 questions on fruit and vegetable consumption from the Clifford et al. (2009) survey [22] to (a) include whole grains, (b) capture consumption in the past month, and (c) include additional portion size examples. Visual aids were provided with each question to aid in portion size estimation. Weekly frequency of (1) cooking or preparing meals, (2) eating out or take-out, and (3) skipping meals in the past month was assessed by asking participants how many times they did each of the above per meal, i.e., for breakfast, lunch, and dinner, separately. Per-meal frequencies were then summed for a total weekly frequency of cooking, eating out, or skipping meals. To assess confidence in including fruits, vegetables, and whole grains in their diet, for each food item students indicated their confidence regarding 10 statements (e.g., “I can find ways to eat fruit at every meal”) using 5-point Likert scales ranging from “extremely confident” to “not at all confident”. To assess cooking confidence, students indicated their confidence on 4 statements (e.g., “I can cook a nutritious meal without spending a lot of money”) using the same Likert scale. Possible total self-efficacy scores ranged from 10 to 50 for food groups and from 4 to 20 for cooking. The full dietary intake questions and self-efficacy scales are listed in Table S2 and Table S3, respectively, of the Supplementary Materials.

Sociodemographic information collected on the baseline survey included age, year in college, gender identity, ethnic/racial identity, living arrangement, food security, receipt of SNAP benefits in the previous 12 months, and whether the participant came from a non-traditional student background. Gender identity was assessed using 6 options (male, female, trans man, trans woman, genderqueer/gender non-conforming, or other specified gender), which was later categorized as male, female, or trans/non-binary. Participants indicated their ethnic/racial identity by selecting 1 or more of 7 categories, which were then classified as Hispanic/Latino/a/e, non-Hispanic White, non-Hispanic Asian, and non-Hispanic Other, due to small numbers of participants in some ethnic/racial groups. Students indicated their living arrangement by selecting from 8 descriptors, which were grouped into 3 categories including on-campus, off-campus, or co-op student housing. Participants were considered to have a non-traditional student background if they responded that they were a re-entry student, first-generation student, current or former foster youth, parent, Pell Grant recipient, or if they had been formerly incarcerated.

Food security was assessed using the Six-Item U.S. Household Food Security Survey Module [23] (September 2012 version [24]), with minor adaptations for the online survey format. A total food security score was created based on the number of affirmative responses to the 6 items and categorized as “food secure” (scores of 0–1), “low food security” (scores of 2–4), or “very low food security” (scores of 5–6) [24]. The latter 2 categories were then collapsed into one category of “food insecure” to create a dichotomous variable for analysis.

### 2.5. Statistical Analyses

Descriptive statistics were used to describe the sample characteristics. Normality of continuous data was assessed based on graphical methods (e.g., histograms), shape parameters (e.g., skewness) and statistics (e.g., Shapiro–Wilk test statistic). Since enrollment in the PFS course (intervention) may have been influenced by participant characteristics, we used a Kruskal–Wallis test (e.g., age) or Chi-square test (e.g., gender) to determine whether participant baseline characteristics differed between groups. As expected for a non-randomized intervention study, baseline differences between the intervention and comparison groups were detected. Therefore, we used propensity score methods to achieve comparability of treated (intervention) and nontreated (comparison) groups in terms of their measured baseline covariates and, in that way, control for confounding in estimating treatment effects [25]. Propensity scores are the probability of being in the intervention group conditional on observed baseline characteristics. This approach generates a balancing score; that is, conditional on the PS, the distribution of observed baseline covariates is similar between intervention and comparison subjects [26]. We estimated propensity scores using a logistic regression model, in which group status was regressed on the baseline characteristics that were significantly (*p* < 0.05) different by group (i.e., gender, race/ethnicity, non-traditional student and Supplemental Nutrition Assistance Program [SNAP] recipient status). We also included food security status in the estimation of propensity scores because enrollment in the course prioritized students at risk of food insecurity. Propensity score weighting was the chosen analytic approach. Specifically, we applied the Average Treatment Effect on the Treated (ATT) weights of the propensity score when estimating the intervention effects to address confounding. Balance diagnostics for the adequacy of the specification of the propensity score model included standardized mean (prevalence) differences for baseline variables between intervention and comparison subjects in the weighted sample, and side-by-side boxplots comparing the distribution of baseline variables between groups in the weighted sample [27].

Before conducting outcome analyses, outliers’ outcome values were investigated and, if needed, truncated at ±4 SD from the mean. Mixed model weighted analyses were conducted with subject as the random effect to account for repeat measurements, group (i.e., comparison and intervention), and time point (i.e., pre and post) as fixed effects, and an interaction term of time point × group to test the statistical significance of any between-group differences in the change in the outcome between the pre and posttest. Each study outcome was modeled separately, and ATT weights were applied in each regression model. Mixed linear regression was used to analyze normally distributed data (e.g., self-efficacy scores), and mixed Poisson regression was applied for analysis of count data (e.g., daily servings of fruits). Least squares means and standard errors were calculated for each time point (i.e., pre and post) and group (i.e., comparison and intervention) and, in the case of the Poisson models, were exponentiated to obtain mean counts. Beta coefficients for each interaction term and corresponding *p*-values were also included, as these coefficients represent the Difference-in-Difference (DiD) estimates [28], that is,

$$DiD=({Mean}_{IE}-{Mean}_{IB})-({Mean}_{CE}-{Mean}_{CB})$$

where I refers to intervention (or treatment) group, C denotes the comparison group, B indicates baseline, and E refers to the endline. Hypothesis testing was two-sided, and the significance level was set to 5%. All statistical analyses were conducted using SAS version 9.4.

## 3. Results

Across the four semesters, 194 students enrolled (and remained enrolled) in the PFS course and were invited to participate in the study. Of these, 95 (49%) consented, completed the baseline survey, and were enrolled in the study. The comparison group comprised 115 students from the general undergraduate student population who completed the baseline survey, after removing one participant who had previously taken the PFS course. In total, four participants in this group enrolled in the study during more than one semester; in these cases, either the first semester of participation (*n* = 3) or the semester in which they participated more fully (*n* = 1) was included.

Of all the participants in the baseline sample (*n* = 210), 129 (61%), including 50 (53%) of course enrollees and 79 (69%) of comparison group participants, completed an endline survey and had data available for at least one of the primary outcomes. The sociodemographic characteristics of these participants, who were considered in the propensity score analysis, are described in Table 1. A majority of participants were female (76%), of Asian ethnicity (56%), in their junior or senior year (69%), and lived in off-campus housing (72%). About half of all participants reported coming from a non-traditional student background, with 43% being first-generation students and 36% receiving Pell Grants, and half reported experiencing food insecurity (Table 1). More students in the comparison group identified as White (23%) compared to the intervention group (8%), while more PFS-enrolled students reported non-traditional student status (71% vs. 42%, *p* = 0.001) and that they had received SNAP benefits in the previous 12 months at baseline (45% vs. 22%, *p* = 0.005).

In the final weighted sample, which included 121 individuals due to missing covariate data (*n* = 4) and propensity scores falling outside of the support region (*n* = 4), standardized mean differences in the included covariates were less than 0.10. With regard to the study outcomes at baseline, between-group testing from the mixed effects linear or Poisson regression models revealed differences in several outcomes, such as lower self-efficacy for consuming vegetables (*p* = 0.04), lower cooking self-efficacy (*p* = 0.01) and frequency (*p* = 0.03), and a higher frequency of skipping meals (*p* = 0.01) among PFS course participants.

At the end of the semester, students enrolled in the PFS course reported more positive changes in self-efficacy (scores) with regard to consuming fruit (group × time β-coefficient [SE]: 6.33 [1.19]), vegetables (5.43 [1.42]), whole grains (5.83 [1.40]), and cooking (3.25 [0.57]) compared to students in the comparison group, who reported slight decreases in these outcomes from baseline to endline, with the exception of cooking self-efficacy (Table 2). In addition, Poisson models indicated participation in the PFS course was associated with smaller pre–post decreases in reported consumption of vegetables (0.35 [0.16]) compared with non-enrolled individuals and with increased cooking frequency (0.22 [0.10]), while no associations were found with the consumption of fruit (0.05 [0.17]) or whole grains (0.16 [0.12]) (Table 3). Course participation was associated with a relative decrease (−0.47 [0.16]) in the frequency of skipping meals in the past month compared to non-participation, but not with the frequency of eating out or take out (0.09 [0.17]) (Table 3).

## 4. Discussion

This study finds that college students who participated in an elective course aimed at improving food and cooking skills through a combined lecture and hands-on cooking class format experienced significant improvements in self-efficacy and behaviors related to cooking and healthy eating, relative to a comparison group. Course participants reported more positive changes in cooking confidence and self-efficacy around consuming fruit, vegetables, and whole grains than students who did not take the course, confirming the findings of our previous study, which relied on an analysis of pre–post changes [12]. We also observed improvements related to course participation versus non-participation in the reported frequency of cooking and skipping meals, in line with our previous study. However, course participation was unrelated to the consumption of fruit and whole grains. While both groups reported average decreases in vegetable consumption from baseline to endline, the decrease in the intervention group was 41% (exponentiated group × time β = 1.41) smaller than in the weighted comparison group.

Our finding of improved self-efficacy around cooking and healthy eating in response to the PFS course is consistent with previous research. Two systematic reviews, together covering studies through 2016, found that adults’ confidence and knowledge around healthy eating often increased after participation in cooking and nutrition interventions [16,17]. Since then, a small number of studies have evaluated the impact of cooking interventions including a teaching kitchen component in college students [12,18,19,20]. These studies observed increases in self-efficacy in cooking and fruit and vegetable consumption [12,18], nutrition or cooking knowledge [18,19], or Cooking and Food Provisioning Action Scale (CAFPAS) scores [20] among participants. The current study contributes additional evidence of the potential effectiveness of food self-efficacy curricula with hands-on cooking experiences, in particular in an ethnically diverse sample of college students, many of whom came from non-traditional student backgrounds (e.g., first generation) and were at a higher risk of food insecurity. Through this work and our previous study [12], we also add to the literature preliminary evidence that the potential impacts on healthy eating self-efficacy include increased confidence in consuming whole grains. However, it should be noted that the scales used to assess whole grains consumption and self-efficacy were not specifically validated; although, they were based on similar scales validated for fruits and vegetables by Clifford et al. [22].

While the current literature, including the results presented here, provides evidence that cooking interventions are effective in increasing confidence and skills (i.e., self-efficacy) around healthy meal preparation and consumption, associated changes in behavior have not been consistently demonstrated [18,19,20]. In a randomized and controlled trial evaluating the impact of a 6-week cooking skills intervention among university students in Brazil, improvements in self-efficacy, which were maintained at 6 months following the intervention, did not translate into more frequent home cooking postintervention or at follow up [18]. Further, decreases in participants’ consumption of fast food and eating in snack bars observed postintervention were not sustained after 6 months [18]. In another, partially randomized, 4-arm trial, college students living off campus participated in a 6-week cooking class intervention with curricula focused on food agency concepts [20]. At the end of the intervention, pre–post changes in students’ healthy eating index scores did not differ between participants in the cooking class arms vs. the non-cooking class arms, and effects on cooking frequency were inconsistent between the two groups receiving cooking classes [20]. Here, we report somewhat more promising effects, with PFS course participants experiencing more positive changes relative to nonparticipants on several behaviors, including cooking frequency, meal skipping, and vegetable consumption; however, other behaviors were not related to the intervention. These more positive results may be attributable to the longer intervention time of 14 weeks, compared to the 6-week intervention time that characterized the two studies described above. Importantly, the cooking labs, which formed a core part of the PFS course studied here, incorporated several key experiential drivers of behavior change that have been identified in culinary education research [29], namely by providing weekly opportunities for the development and reinforcement of practical skills in an environment that facilitated peer support and collaboration. Additionally, the preparation of new recipes and/or the completion of cooking challenges every week offered opportunities for the experience of challenge, success, and celebration, three other elements identified as important behavior change drivers that may help explain the impacts of successful cooking interventions [29].

In interpreting the study findings, it should be considered that baseline differences existed between groups in several outcomes; thus, improved trajectories (direction and/or magnitude of change) did not necessarily result in the intervention group having better endline outcomes in absolute terms. Further, it is important to note that the endline measurements taken in this study occurred at the end of the semester, a time of heightened stress for college students [30,31] who are often studying and completing end-of-term assignments on irregular and late-night schedules. These circumstances could lead to increased emotional eating and leave less time for home meal preparation. Indeed, lower diet quality marked by a reduced consumption of fruit and vegetables and higher consumption of fast food has been observed in university students during the exam period, in particular for individuals reporting higher stress levels or emotional eating [32]. Thus, this heightened end-of-semester climate may help to explain our observation that, overall, vegetable consumption decreased from the beginning to the end of the semester. However, our results suggest that the PFS course may have been helpful in mitigating decreased vegetable consumption in the intervention group during the final exam period, as seen by the smaller decrease in consumption in this group. Fruit consumption was not an area of emphasis in the course curriculum, which possibly explains the lack of association with this outcome. Overall, the results of this study suggest that a semester-long course targeting the development of practical food knowledge, skills, and strategies with hands-on cooking labs may have an impact on college students’ ability to overcome financial, time-related, or social constraints on cooking and healthy eating.

This study improves upon previous research by the authors, by including a comparison group to isolate changes that were likely related to course participation. Other strengths of our study include the use of survey items that were previously validated for use in college students (or adaptations thereof, in the case of whole grain scales); data collected over multiple semesters, which may help balance out seasonal influences on food consumption or other external events that could vary across years; and the employment of a propensity score weighting approach to reduce potential confounding by baseline characteristics. This study also included a population with a potentially greater need for educational opportunities that target practical strategies for overcoming negative social determinants of food security, cooking, and healthy eating. Compared to previous studies evaluating cooking interventions among college students, most of which were conducted in primarily White [18,20] or non-diverse [13] U.S. or Brazilian samples, the majority of participants in the current study were students of color and students coming from nontraditional backgrounds, such as first-generation college students. Nevertheless, greater representation of particular groups, including Native American and Black or African American students, is needed in future research.

Several limitations are important to note in interpreting these findings. First, despite the use of propensity scores to balance baseline characteristics between the intervention and comparison groups, the intervention was not randomly assigned; thus, residual or unmeasured confounding cannot be ruled out. Baseline differences between the groups existed for some outcomes, such as cooking self-efficacy and frequency, which may point to other unmeasured differences between the groups. For example, information on previous exposure to other nutrition or cooking classes was not collected and could have contributed to between-group differences in outcomes at baseline. Further, previous or concurrent exposure to other nutrition curricula in the intervention or comparison group during the intervention period could have confounded or diluted observed associations with the intervention. Second, selection bias may have been introduced by the low enrollment rate among course participants (49%), convenience sampling of the comparison group, and limited responses to the endline survey and/or main outcomes (61% of the baseline sample). In addition, most study participants (76%) identified as female. Thus, findings may not be generalizable to the general population of college students at risk of food insecurity or those experiencing a high degree of social/structural barriers to participation. A third limitation was that dietary consumption was assessed using self-reported measures, which are subject to recall bias and random measurement error. Similarly, self-reported self-efficacy and behavior measures may have been influenced by social desirability bias, in particular among course participants at the end of the semester; however, the use of self-administered online surveys likely mitigated this to some extent. While the present results suggest that the PFS course may have led to improvements (compared to non-participation) in several outcomes by the end of the course, its potential impact over the long term is unknown, suggesting future intervention studies with additional follow-up measurements are warranted.

## 5. Conclusions

Participation in a semester-long PFS course with a teaching kitchen was associated with improvements in self-efficacy related to cooking and healthy eating among undergraduate students. Compared to students not enrolled in the course, course participants had improved trajectories in the frequency of cooking, frequency of skipping meals, and consumption of vegetables, but not in fruit or whole grain consumption or frequency of eating out. These results suggest the addition of a teaching kitchen lab and content focused on practical skills to college-level nutrition courses may help undergraduate students overcome challenges to healthy eating as they navigate the transition to adulthood; however, whether such a course leads to long-term behavior changes is unknown. Future studies would benefit from using random allocation to the intervention with a delayed intervention approach, as well as follow-up measurements to understand the sustainability of the course’s impact.

## Figures and Tables

**Table 1:** Baseline characteristics of undergraduate students with available outcome data ^1^.

	Intervention (*n* = 50) ^2^	Comparison (*n* = 79) ^2^	Total (*n* = 129) ^2^	*p* ^3^
**Age, y [Median (Q1, Q3)]**	20 (19, 21)	20 (19, 21)	20 (19, 21)	0.76
**Gender**				0.19
Female	34 (69.4)	63 (79.7)	97 (75.8)	
Male	13 (26.5)	11 (13.9)	24 (18.8)	
Trans or non-binary	2 (4.1)	5 (6.3)	7 (5.5)	
**Race/Ethnicity**				0.04
Hispanic/Latino/a/e	15 (30.6)	15 (19.2)	30 (23.6)	
NH White	4 (8.2)	18 (23.1)	22 (17.3)	
NH Asian	30 (61.2)	41 (52.6)	71 (55.9)	
NH Other	0 (0.0)	4 (5.1)	4 (3.1)	
**Undergraduate Year**				0.62
Freshman	9 (18.0)	13 (16.5)	22 (17.1)	
Sophomore	8 (16.0)	10 (12.7)	18 (14.0)	
Junior	14 (28.0)	31 (39.2)	45 (34.9)	
Senior	19 (38.0)	25 (31.6)	44 (34.1)	
**Housing Situation**				0.26
Off-campus	39 (79.6)	53 (67.1)	92 (71.9)	
On-campus	9 (18.4)	21 (26.6)	30 (23.4)	
Co-op	1 (2.0)	5 (6.3)	6 (4.7)	
**Non-Traditional Student ^4^**	35 (71.4)	33 (42.3)	68 (53.5)	0.001
**Received SNAP**	22 (44.9)	17 (21.5)	39 (30.5)	0.005
**Visited Campus Pantry**	18 (36.7)	37 (46.8)	55 (43.0)	0.51
**Food Insecurity**	27 (56.3)	36 (46.2)	63 (50.0)	0.27

^1^ Categorical data are presented as count (%), unless otherwise specified. ^2^ Sample sizes vary among some variables due to missing data. ^3^ *p*-values are from Kruskal–Wallis or Chi-square tests for continuous and categorical data, respectively. ^4^ Non-traditional student status is defined as re-entry or first-generation students, current or former foster youth, students who were formerly incarcerated, parents, or Pell Grant recipients. Abbreviations: NH, Non-Hispanic; SNAP, Supplemental Nutrition Assistance Program.

**Table 2:** Association between participation in a personal food skills course with a teaching kitchen and self-efficacy in cooking and consuming fruit, vegetables, and whole grains ^1^.

	*N*	Intervention		Comparison		*p* ^2^
		Baseline	Endline	Baseline	Endline	
Fruit self-efficacy score	117	32.77 (1.04)	37.13 (1.04)	34.74 (0.99)	32.78 (0.99)	<0.0001
Vegetable self-efficacy score	116	33.74 (1.07)	38.34 (1.07)	36.89 (1.05)	36.06 (1.05)	0.0002
Whole grain self-efficacy score	114	35.76 (1.18)	41.26 (1.18)	38.58 (1.14)	38.25 (1.14)	<0.0001
Cooking self-efficacy score	115	11.76 (0.46)	16.24 (0.46)	13.45 (0.44)	14.69 (0.44)	<0.0001

^1^ Least squares means (SE) from mixed effects model using propensity-score-weighted data are presented. ^2^ *p* values are for the group × time point interaction term.

**Table 3:** Association between participation in a personal food skills course with a teaching kitchen and usual consumption of fruit, vegetables, and whole grains and meal preparation behaviors in the past month ^1^.

	*N*	Intervention		Comparison		*p* ^2^
		Baseline	Endline	Baseline	Endline	
Fruit (cups/day)	121	2.40 (0.35)	1.71 (0.26)	2.67 (0.36)	1.81 (0.26)	0.77
Vegetables (cups/day)	121	2.39 (0.35)	1.99 (0.30)	3.34 (0.44)	1.96 (0.28)	0.03
Whole grains (ounces/day)	120	4.85 (0.59)	3.80 (0.48)	5.65 (0.61)	3.77 (0.43)	0.18
Cooking (meals/week)	113	5.86 (0.76)	6.64 (0.85)	8.68 (0.98)	7.87 (0.90)	0.03
Skipping (meals/week)	102	4.65 (0.54)	3.56 (0.44)	3.02 (0.37)	3.71 (0.44)	0.003
Eating out/take-out (meals/week)	106	3.38 (0.36)	3.44 (0.37)	3.15 (0.35)	2.92 (0.33)	0.58

^1^ Exponentiated least squares means (SE) from mixed effects Poisson regression using propensity-score-weighted data are presented. ^2^ *p* values are for the group × time point interaction term.

## Data Availability

The data presented in this study is not available due to ethical approval restrictions.

## Supplementary Materials

The following supporting information can be downloaded at: https://www.mdpi.com/article/10.3390/nu16050585/s1. Table S1: Screening questions used by the instructor to prioritize students with greater need for admittance into the personal food skills course; Table S2: Survey questions used to assess usual consumption of vegetables, fruit, and whole grains; Table S3: Scale items used to assess self-efficacy in cooking and incorporating fruits, vegetables, and whole grains into the diet.
