# Supplementary material for: Impact of a Food Skills Course with a Teaching Kitchen on Dietary and Cooking Self-Efficacy and Behaviors among College Students
Source: Nutrients. 2024 Feb 21;16(5):585. doi: 10.3390/nu16050585 (PMC10933768; doi:10.3390/nu16050585)
Supplement: Supplementary file 1 [file nutrients-16-00585-s001.zip › nutrients-2845074-supplementary.pdf]

**Supplementary Tables**

French et al. Impact of a food skills course with a teaching kitchen on dietary and cooking self-efficacy and behaviors among college students.

**Table S1.** Screening questions used by the instructor to prioritize students with greater need for admittance into the personal food skills course

| No. | Question |
|---|---|
| 1 | Why do you want take this course, which teaches students how to prepare and buy affordable and healthy foods to prevent or manage food insecurity and promote health? |
| 2 | What experience, if any, do you have with purchasing and preparing your own food? |
| 3 | Do you belong to any of the CE3 [1] programs on campus? (EOP, Hope Scholars, Underground Scholars, etc.) If so, which program? |
| 4 | Are you a CalFresh recipient? [2] |

[1] The Centers for Educational Equity and Excellence (CE3) comprise several programs and centers at the study site university that provide services to systemically marginalized students with the aim of empowering students to achieve and lead.

[2] For the 2022 Spring semester, this question was worded as follows: "Are you a recipient of CalFresh Benefits?"
Abbreviations: CE3, Centers for Educational Equity and Excellence; EOP, Educational Opportunity Program.

**Table S2.** Survey questions used to assess usual consumption of vegetables, fruit, and whole grains [1]

| Construct | Question |
|---|---|
| Usual vegetable consumption in the past month | About how many cups of vegetables did you eat per day during the past month? For reference, one cup is about the size of a baseball. Examples of 1 cup of vegetables include 1 cup of cooked leafy greens, 2 cups of lettuce or raw greens, 12 baby carrots, or 1 large raw tomato. |
| Usual fruit consumption in the past month | On average, how many cups of fruit did you eat per day during the past month? For reference, one cup is about one small piece. Examples of 1 cup of fruit include 1 small apple, 1 large banana, 1 cup (8 oz.) of 100% juice or canned fruit, or ½ cup of dried fruit. |
| Usual whole grains consumption in the past month | On average, how many ounces of whole grains did you eat per day during the past month? For reference, one ounce is equal to: one slice of whole wheat bread, one 1" mini bagel, ½ English muffin, 5 whole wheat crackers, 1 cup of breakfast cereal, ½ cup of cooked rice/ pasta/oatmeal, 1 small flour tortilla (6" diameter), or 3 cups of (popped) popcorn. |

[1] Questions adapted from the personal factors survey in Clifford *et al* (2009) [1].

**Table S3.** Scale items used to assess self-efficacy in cooking and incorporating fruits, vegetables, and whole grains into the diet [1]

| Construct | Scale Items [2] |
|---|---|
| Self-efficacy including fruits, vegetables, and whole grains in the diet | 1. I can include *[fruit / vegetables / whole grains]* into my diet when I'm on a limited budget.<br>2. I can eat at least 1 cup of *[fruit / vegetables / whole grains]* daily.<br>3. I can eat at least 2 cups of *[fruit / vegetables / whole grains]* daily.<br>4. I can eat at least 3 cups of *[fruit / vegetables / whole grains]* daily.<br>5. I can keep *[fruit / vegetables / whole grains*] at hand/readily available.<br>6. I can shop for a variety of *[fruit / vegetables / whole grains]*.<br>7. I can make time to eat *[fruit / vegetables / whole grains]*.<br>8. When I eat at home, I can eat more *[fruit / vegetables / whole grains]*.<br>9. I can find ways to eat *[fruit / vegetables / whole grains]* at every meal.<br>10. I can select *[fruit / vegetables / whole grains]* at the grocery store. |
| Cooking self-efficacy | 1. I can cook a nutritious meal.<br>2. I can cook a meal in a short amount of time.<br>3. I can cook a nutritious meal without spending a lot of money.<br>4. I can follow a recipe. |

[1] Questions adapted from survey items developed by Clifford *et al* (2009) [1].

[2] Each item was assessed using a 5-item Likert scale including "Exremely confident", "Very confident", "Moderately confident", "Not very confident", and "Not at all confident".

## Reference


1. Clifford, D.; Anderson, J.; Auld, G.; Champ, J. Good Grubbin': impact of a TV cooking show for college students living off campus. *Journal of nutrition education and behavior* **2009**, *41*, 194-200.
